# Rapidly Progressive Fulminant Perimyocarditis Requiring Early Venoarterial Extracorporeal Membrane Oxygenation (VA-ECMO) Support in a Previously Healthy Young Adult

**DOI:** 10.7759/cureus.107404

**Published:** 2026-04-20

**Authors:** Jeffrey Hamada, Lauren McCormack, Richard Chang, Eric H Chou

**Affiliations:** 1 Emergency Medicine, Baylor Scott & White All Saints Medical Center, Fort Worth, USA; 2 Medicine, Baylor Scott & White All Saints Medical Center, Fort Worth, USA; 3 Emergency Medicine, Taipei Hospital, Ministry of Health and Welfare, New Taipei City, TWN

**Keywords:** ecmo, fulminant myocarditis, myocarditis, perimyocarditis, va-ecmo

## Abstract

Fulminant perimyocarditis is a rare but life-threatening inflammatory cardiomyopathy that can rapidly progress to cardiogenic shock. Early recognition and timely initiation of mechanical circulatory support are critical for survival. We report the case of a previously healthy young male who initially presented with chest pain and dyspnea and was treated for presumed pericarditis in the setting of a possible infectious process. He rapidly deteriorated with hypotension, hypoxia, and rising lactate, consistent with cardiogenic shock. Transthoracic echocardiography demonstrated reduced left ventricular systolic function, and coronary angiography revealed non-obstructive coronary arteries. The patient was emergently supported with venoarterial extracorporeal membrane oxygenation (VA-ECMO) and Impella (Johnson & Johnson MedTech, New Brunswick, NJ, USA) for left ventricular unloading, along with inotropic support and high-dose corticosteroids. Hemodynamic assessment demonstrated elevated filling pressures despite apparently normal cardiac output, consistent with mechanically supported circulation. Endomyocardial biopsy confirmed acute myocarditis. The patient improved rapidly, allowing discontinuation of mechanical circulatory support by hospital day three and discharge by day nine. At one-month follow-up, he had returned to baseline functional status. This case highlights the rapid progression of fulminant perimyocarditis and supports early initiation of VA-ECMO as a bridge to recovery in refractory cardiogenic shock. The observed discordance between structural recovery and long-term functional outcomes underscores the importance of ongoing follow-up. Further studies are needed to define optimal management strategies and the role of adjunctive therapies.

## Introduction

Acute pericarditis is an inflammatory syndrome of the pericardial sac that typically presents with sharp pleuritic chest pain that worsens with inspiration and improves when sitting forward. In Western populations, its annual incidence is estimated at about 28 cases per 100,000 persons, with approximately three per 100,000 requiring hospital admission [1,2]. Although many cases are labeled idiopathic, this designation often reflects presumed viral disease after exclusion of other causes; recognized etiologies also include bacterial and other infections, autoimmune and rheumatologic disorders, malignancy, post-cardiac injury syndromes, and drug-related inflammation [2-4].

Myocardial involvement may accompany pericarditis and can be suggested by elevated cardiac biomarkers, arrhythmias, electrocardiographic abnormalities, or imaging evidence of myocardial inflammation. The terms myopericarditis and perimyocarditis are related but not synonymous. Authoritative definitions distinguish them primarily by ventricular function: myopericarditis describes a pericarditis-predominant presentation with evidence of myocardial injury but preserved left ventricular systolic function, whereas perimyocarditis refers to myocarditis-predominant disease with associated pericardial inflammation and demonstrable ventricular dysfunction or regional wall motion abnormalities [1,5]. Although terminology may vary across studies, ventricular function remains the key distinguishing feature. Viruses remain the most frequently implicated infectious causes of myocarditis, although the dominant pathogen varies by era, geography, and diagnostic method. Historically, adenovirus and enteroviruses were commonly reported, while more recent series have also identified parvovirus B19 and human herpesvirus 6 [4,6].

Overall, most cases of pericarditis, including those with limited myocardial involvement, follow a benign course with recovery of ventricular function [5,7]. However, a small subset progresses to fulminant myocarditis with severe biventricular dysfunction, malignant arrhythmias, and cardiogenic shock. Fulminant presentations are uncommon, accounting for approximately 3%-9% of acute myocarditis cases, and are characterized by rapid onset of severe myocardial dysfunction with cardiogenic shock, life-threatening arrhythmias, and potential multiorgan failure [3,4,8-10]. In these critically ill patients, venoarterial extracorporeal membrane oxygenation (VA-ECMO) may provide lifesaving temporary circulatory support as a bridge to myocardial recovery [9-11]. Although evidence remains limited and no universally accepted criteria exist for ECMO initiation in this setting, small series have reported encouraging survival, including hospital discharge in seven of nine patients in one study [12].

This report describes a case of fulminant perimyocarditis with rapid progression in a previously healthy young adult, successfully managed with early VA-ECMO support. It underscores the importance of early recognition and timely intervention in fulminant disease.

## Case presentation

A previously healthy 26-year-old male presented to the emergency department (ED) with a three-day history of progressive chest pain and dyspnea. Several days prior, he had been evaluated at an outside hospital, where he was treated for presumed bacterial pneumonia and probable pleuritis. His chest pain was left-sided, radiating to the left jaw, sharp in quality, and both pleuritic and positional, worsening when supine or lying on his left side and improving with sitting upright and leaning forward. He denied prior similar symptoms. His medical, surgical, and family histories were unremarkable. He denied alcohol or recreational drug use; however, collateral history later revealed use of vape pens and nicotine salts.

**Figure 1:**
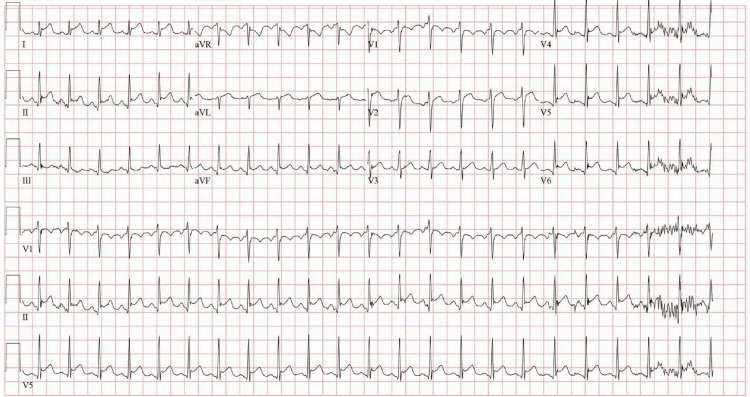
Initial EKG demonstrating diffuse ST elevations with ST depression in lead aVR, suggestive of acute pericarditis

**Figure 2:**
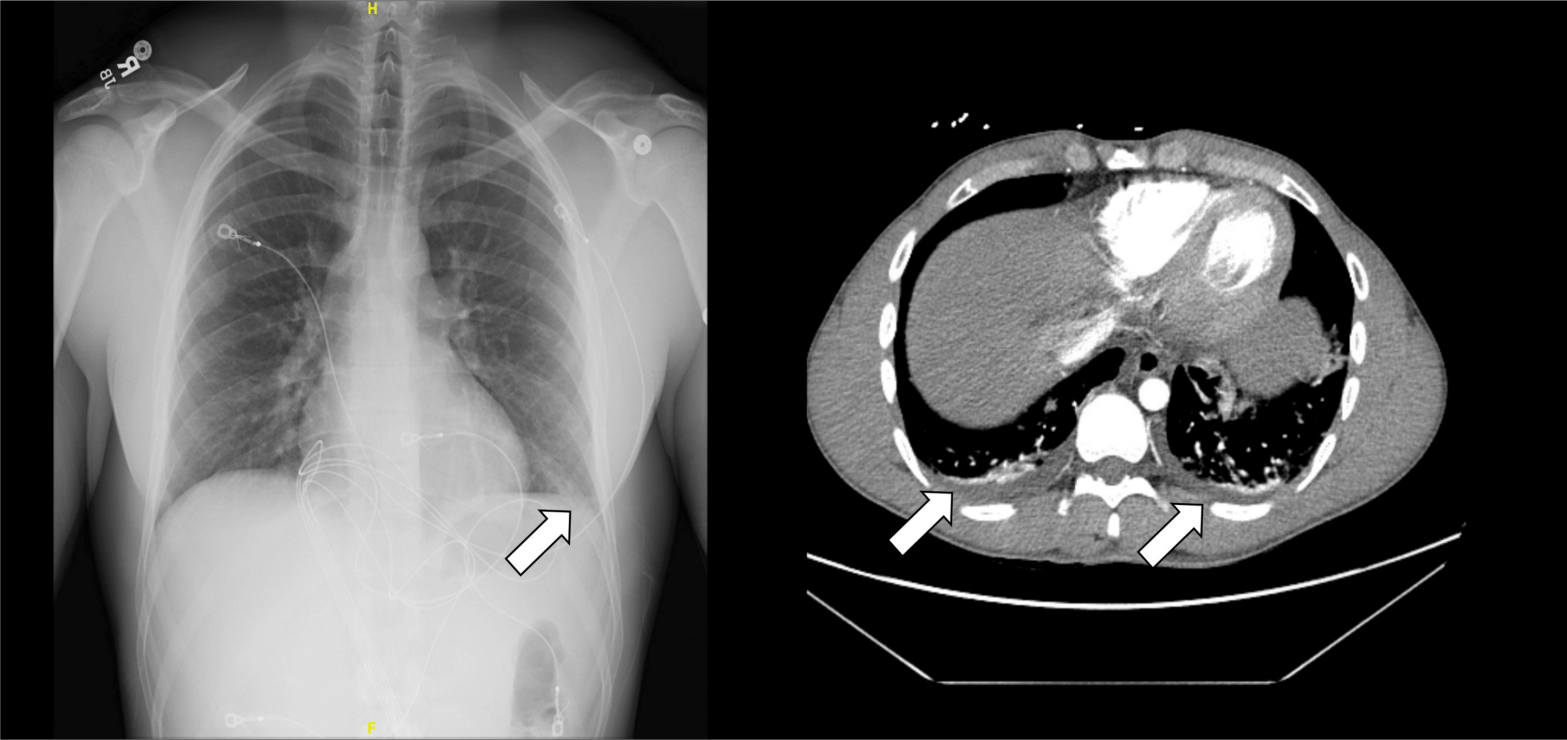
Initial chest radiograph and computed tomography angiography (CTA) of the chest demonstrating a subtle lower lobe infiltrates (arrows).

On presentation, vital signs were notable for a heart rate of 101 beats per minute, with otherwise normal temperature (36.9 °C), blood pressure (119/78 mmHg), respiratory rate (20 breaths per minute), and oxygen saturation (100% on room air). Electrocardiogram demonstrated diffuse ST-segment elevations with reciprocal ST depression in lead aVR (Figure 1). Laboratory studies revealed a markedly elevated high-sensitivity troponin I of 31,097 ng/L (reference <59 ng/L), leukocytosis of 16.5 ×10³/µL (reference 4.0-10.0 ×10³/µL), and mildly elevated liver enzymes (Table 1). Chest radiography demonstrated bilateral interstitial infiltrates. Computed tomography angiography of the chest showed no evidence of pulmonary embolism but revealed mild patchy bilateral pulmonary infiltrates, most pronounced in the right lower lobe, along with mild dependent atelectasis and small bilateral pleural effusions. These findings were nonspecific and could represent an inflammatory or infectious process (Figure 2). An extensive infectious workup, including testing for viral pathogens (e.g., Coxsackie virus and COVID-19), urinary infection, and bacteremia, was unrevealing. The patient was treated symptomatically with nonsteroidal anti-inflammatory drugs and opioids and was started empirically on intravenous vancomycin and ceftriaxone for presumed multifocal pneumonia based on clinical and imaging findings.

**Table 1:** Summary of quantitative laboratory investigations obtained at ED presentation. Abbreviations: ED, Emergency Department; AST, aspartate aminotransferase, ALT, alanine aminotransferase, BNP, B-type natriuretic peptide.

Laboratory parameter	ED visit	Reference range
White blood cell count (×10³/µL)	16.5	4.0–10.0
Hemoglobin (g/dL)	12.4	12.0–16.0
AST (U/L)	130	15-37
ALT (U/L)	130	16-6
Bilirubin, Total (mg/dL)	0.9	0.0-1.1
Troponin (ng/L)	31,097	<59
BNP (pg/mL)	371	5-100
C-Reactive Protein (mg/dL)	24.7	0.0-0.3
Sedimentation Rate (ESR, mm/h)	61	0-15

While awaiting admission, the patient developed rapid clinical deterioration with worsening hypoxia (SpO₂ 92% on supplemental oxygen), hypotension (91/49 mmHg), and tachycardia (heart rate 140 bpm). Transthoracic echocardiography demonstrated a normal-sized left ventricle with mildly reduced systolic function (ejection fraction 43%) and global hypokinesis. Global longitudinal strain was markedly reduced at −9.6% (normal <−18%), consistent with diffuse myocardial dysfunction. A trivial circumferential pericardial effusion was present without evidence of tamponade physiology. Despite escalating vasopressor support, metabolic acidosis worsened, with lactate rising from 1.6 to 3.3 mmol/L (reference 0.9-1.7 mmol/L), consistent with cardiogenic shock. The patient was emergently cannulated for VA-ECMO for hemodynamic support. Impella CP (Johnson & Johnson MedTech, New Brunswick, NJ, USA) was placed for left ventricular unloading in the setting of reduced systolic function and hemodynamic compromise. Coronary angiography demonstrated non-obstructive coronary arteries. High-dose intravenous methylprednisolone (500 mg twice daily) was initiated for suspected inflammatory perimyocarditis, and inotropic support with milrinone was started.

Hemodynamic assessment under VA-ECMO (flow ~4.6 L/min; typical adult range ~3-5 L/min) with Impella support demonstrated elevated filling pressures, including right atrial pressure 14 mmHg (reference 2-8), pulmonary capillary wedge pressure 20 mmHg (reference 6-12), and left ventricular end-diastolic pressure 17 mmHg (reference 5-12). Pulmonary artery pressure was within normal limits (mean 23 mmHg; reference 10-20) with low pulmonary vascular resistance (<1 Wood unit; reference <3). Fick cardiac output and index were 5.3 L/min and 2.8 L/min/m² (reference 4-8 and 2.5-4.0), reflecting mechanically supported flow. Coronary angiography demonstrated non-obstructive disease. Overall, findings are consistent with persistent primary myocardial dysfunction despite mechanical circulatory support.

Further evaluation for infectious causes, including testing for methicillin-resistant Staphylococcus aureus, hepatitis A, B, and C, West Nile virus, HIV, and enterovirus, was negative. Drug-induced myocarditis was considered unlikely given a negative toxicology screen and limited medication exposure. Endomyocardial biopsy performed on hospital day three demonstrated nonspecific findings consistent with acute myocarditis without a definitive etiology. 

The patient exhibited rapid clinical improvement. Mechanical circulatory support was weaned quickly, with removal of Impella support after one day and successful decannulation from VA-ECMO by hospital day three. Follow-up echocardiography demonstrated recovery of left ventricular function with an ejection fraction of 50-55% and resolution of global hypokinesis. Antibiotics were narrowed to doxycycline and discontinued after a seven-day course in the absence of an identified bacterial source. Over a six-day intensive care unit course, the patient was weaned off vasopressors and maintained adequate oxygenation on room air. He was subsequently transferred to the general medical floor and continued to improve with rehabilitation therapies.

The patient was discharged home after nine days of hospitalization without the need for additional support services. At discharge, he was prescribed lisinopril 10 mg daily for persistent hypertension and continued on a corticosteroid taper beginning with prednisone 60 mg daily, with outpatient cardiology follow-up to guide further tapering. At follow-up eight days post-discharge, cardiac biomarkers had normalized, and N-terminal pro-B-type natriuretic peptide (NT-proBNP) levels had significantly improved. By one month, the patient had returned to baseline functional status, reporting no dyspnea, chest pain, or exercise intolerance, consistent with New York Heart Association (NYHA) class I functional capacity. Functional recovery was further assessed using the Minnesota Living with Heart Failure Questionnaire (MLHFQ), which demonstrated a score of 43 at eight days post-discharge, indicating moderate impairment in quality of life. This improved to a score of 6 at one month, consistent with minimal impact. However, at one-year follow-up, the MLHFQ score increased to 25, suggesting a recurrence of moderate functional limitation. Follow-up cardiac magnetic resonance imaging (MRI) demonstrated no evidence of active myocardial inflammation, with no appreciable myocardial edema or late gadolinium enhancement to suggest fibrosis or infiltrative disease. Biventricular size and function were normal, with a left ventricular ejection fraction of 65% and right ventricular ejection fraction of 66%, and no regional wall motion abnormalities.

## Discussion

We present the case of a previously healthy young male initially diagnosed with pericarditis in the setting of a presumed inflammatory or infectious process. The diagnosis of perimyocarditis was established based on clinical, electrocardiographic, laboratory, and histopathologic findings, including diffuse ST-segment elevations with PR-segment depression, markedly elevated troponin levels, and biopsy-confirmed acute inflammatory myocarditis. The patient rapidly deteriorated in the ED, progressing to fulminant perimyocarditis with cardiogenic shock. Early initiation of VA-ECMO, along with inotropic support and high-dose corticosteroids, resulted in rapid clinical improvement. Mechanical circulatory support was discontinued by hospital day three, and the patient was discharged after nine days with return to baseline functional status by one-month follow-up.

Fulminant perimyocarditis is a rare but severe inflammatory cardiomyopathy characterized by rapid progression to cardiogenic shock [10,13]. Although the underlying mechanism of left ventricular dysfunction differs from ischemic etiologies such as acute coronary syndrome, the initial management of cardiogenic shock remains grounded in standard principles, including optimization of preload, afterload, and contractility with vasopressors and inotropes [10,13]. In this case, rapid hemodynamic deterioration despite medical therapy prompted early escalation to mechanical circulatory support. Although fulminant myocarditis and perimyocarditis are relatively rare, existing literature includes case reports, systematic reviews, and guideline statements describing a range of etiologies and management strategies [2,9,14-16]. Viral causes such as COVID-19, influenza, and post-vaccination syndromes, as well as autoimmune conditions including systemic lupus erythematosus, have all been associated with fulminant presentations [16-19]. Subsequent activation of innate and adaptive immune pathways, including T-cell-mediated cytotoxicity and autoantibody formation, leads to amplified myocardial inflammation, interstitial edema, and myocyte necrosis. Pro-inflammatory cytokines (e.g., TNF-α, IL-1, IL-6) further depress myocardial contractility and promote systemic inflammation. In fulminant cases, this dysregulated immune response results in rapid-onset severe ventricular dysfunction, arrhythmias, and cardiogenic shock, representing the extreme myocarditis-predominant end of the spectrum (i.e., perimyocarditis) [9,13].

Recent observational studies and registry data have demonstrated that patients with fulminant myocarditis requiring VA-ECMO can achieve favorable survival and recovery of cardiac function, particularly when early mechanical circulatory support is initiated in cardiogenic shock [8,10,12,14,15,20]. A systematic review of published cases suggests one-year survival rates ranging from approximately 57% to 78% with mechanical circulatory support [15]. In addition, a scientific statement from the American Heart Association (AHA) supports early consideration of extracorporeal support in fulminant myocarditis [9].

Despite these advances, the evidence base remains limited, with most data derived from small series and observational reports. Consequently, the role of adjunctive therapies, including colchicine and intravenous immunoglobulin, remains uncertain, particularly in fulminant presentations, where management is often prioritized toward rapid hemodynamic stabilization [2,9,10]. The patient’s clinical course supports the role of early VA-ECMO as a bridge to recovery, rather than as delayed rescue therapy. Cannulation occurred shortly after the onset of hypotension, hypoxia, and rising lactate, consistent with evolving cardiogenic shock. This approach aligns with recommendations from the AHA, which emphasize early recognition and timely initiation of mechanical circulatory support in fulminant myocarditis [2,9]. The addition of Impella for left ventricular unloading reflects evolving strategies to reduce ventricular distension and myocardial oxygen demand during extracorporeal support [2,9]. Adjunctive therapies were an important component of management. Inotropic and vasopressor support were used to maintain end-organ perfusion, consistent with standard cardiogenic shock care [2,10]. High-dose corticosteroids were initiated due to the fulminant presentation, absence of obstructive coronary disease, and concern for inflammatory or immune-mediated myocardial injury, although their role in presumed viral myocarditis remains controversial [2,9,10]. Therapies such as colchicine or intravenous immunoglobulin were not utilized, as their benefit in fulminant presentations is not well established and the primary priority was stabilization of hemodynamic compromise.

Hemodynamic data further support a primary myocardial process. Despite apparently normal cardiac output, elevated right- and left-sided filling pressures indicate persistent ventricular dysfunction, with cardiac output values reflecting extracorporeal support rather than intrinsic recovery [2,10]. The combination of low pulmonary vascular resistance and non-obstructive coronary arteries helps exclude pulmonary vascular and ischemic etiologies, reinforcing the diagnosis of inflammatory cardiomyopathy. Longitudinal follow-up highlights important considerations in recovery. Despite normalization of ventricular function and absence of residual inflammation or fibrosis on cardiac MRI, the patient demonstrated partial decline in quality-of-life measures at one year. This discordance suggests that functional recovery may not fully parallel structural recovery and underscores the importance of long-term monitoring beyond imaging alone.

This case contributes to the existing literature on VA-ECMO in fulminant myocarditis; however, its novelty lies in the rapid transition from a nonspecific inflammatory or pulmonary presentation to fulminant perimyocardial shock, necessitating early mechanical support before a definitive diagnosis was established. Importantly, most available data derive from studies of fulminant myocarditis broadly, and extrapolation to perimyocarditis or overlap syndromes remains limited. Several limitations should be acknowledged. As a single case, causal relationships between therapies and recovery cannot be established, and the relative contributions of corticosteroids, inotropes, and mechanical support remain uncertain. Initial viral testing was unrevealing, and repeat viral serology/PCR testing was not performed during hospitalization. This represents a limitation, as false-negative early testing cannot be fully excluded. Although a rheumatologic etiology was considered, further serologic evaluation was not pursued because the biopsy findings were nonspecific, there were no accompanying clinical features strongly suggestive of systemic autoimmune disease, and the patient demonstrated rapid clinical and echocardiographic improvement with supportive care and corticosteroid therapy. Additionally, although cardiac MRI demonstrated no residual inflammation or fibrosis, advanced rhythm monitoring was not performed, limiting assessment of arrhythmic risk. Future research should focus on multicenter registries and prospective studies that distinguish myocarditis, myopericarditis, and perimyocarditis phenotypes. Key priorities include defining optimal timing of VA-ECMO initiation, clarifying the role of adjunctive immunomodulatory therapies, and establishing standardized approaches to long-term surveillance.

## Conclusions

Fulminant perimyocarditis is a rare but life-threatening condition that may initially present with nonspecific symptoms and rapidly progress to cardiogenic shock. This case highlights the importance of early recognition and timely escalation to mechanical circulatory support, particularly VA-ECMO as a bridge to recovery. Despite normalization of cardiac structure and function, persistent or recurrent functional limitations may occur, underscoring the need for longitudinal follow-up. Further research is needed to define optimal management strategies and clarify the role of adjunctive therapies in fulminant inflammatory cardiomyopathies.
